# Safety and effective of laparoscopic microwave ablation for giant hepatic hemangioma: A retrospective cohort study

**DOI:** 10.1016/j.amsu.2019.02.001

**Published:** 2019-02-05

**Authors:** Libo Chen, Lei Zhang, Min Tian, Qinggang Hu, Lei Zhao, Jun Xiong

**Affiliations:** a Department of Emergency Surgery, Union Hospital, Tongji Medical College, Huazhong University of Science and Technology, Wuhan, Hubei Province, 430022, China; b Department of Hepatobiliary Surgery, Union Hospital, Tongji Medical College, Huazhong University of Science and Technology, Wuhan, Hubei Province, 430022, China; c Department of Infectious Disease, Union Hospital, Tongji Medical College, Huazhong University of Science and Technology, Wuhan, Hubei Province, 430022, China

**Keywords:** Liver, Hemangioma, Laparoscopes, Ablation techniques

## Abstract

**Introduction:**

The purpose of this study was to evaluate the advantages and disadvantages of laparoscopic microwave ablation (LMWA) as compared with conventional open resection (ORES) for the treatment of giant hepatic hemangioma.

**Methods:**

and analysis: A retrospective chart review was conduct on patients with hepatic hemangioma underwent LMWA or ORES between 2014 and 2016.

**Results:**

Of 131 patients, 37 patients underwent ORES and 94 patients underwent LMWA. Blood loss, operative time, postoperative hospital stay, hospital cost (RMB) were significantly different between two groups. Patients after LMWA experienced significantly less pain than those patients undergoing ORES. At a mean follow-up period of 12.8 ± 3.6 months in ORES group and 13.5 ± 2.5 months in LMWA group, no long-term complication was observed.

**Conclusion:**

Compared with ORES, LMWA is a safe and effective minimally invasive for treating giant hepatic hemangioma.

## Highlights

• Retrospectively analysed the clinical data of patients with hepatic haemangioma and investigated the efficacy of operation.
• Technical aspects of laparoscopic microwave ablation for giant hepatic haemangioma were listed base on operation experience.

## 1 Introduction

Liver hemangioma is a common benign tumour in the liver, with an incidence rate of 0.4%–20% [1]. Most patients with liver hemangioma do not have self-observable symptoms [2]. However, when lesions reach 5 cm or larger, they are called huge hepatic hemangiomas (giant hemangiomas) [3], and some patients may have symptoms such as abdominal discomfort, jaundice, swelling and thrombocytopenia [4]. Currently, small asymptomatic hemangioma generally does not require treatment, but it should be assessed via an ultrasound test every 3–6 months to dynamically monitor the status of the lesion mass [5,6]. Treatment is needed for symptomatic cases or cases in which the lesion is continuously growing [7]. Classic treatment mainly includes the following: surgical resection, hepatic artery ligation, hepatic artery interventional embolization, radiation therapy and steroid therapy [[8], [9], [10]]. Some patients with hepatic hemangioma even need a liver transplant [11,12].

Surgical resection is the most effective treatment option for patient with hemangioma, but it is a highly invasive procedure. Several authors have reported that the morbidity and mortality rates of surgical resection for hemangioma were up to 27% and 3%, respectively [13,14]. Minimally invasive procedures such as laparoscopic hepatectomy, hepatic artery ligation, hepatic artery interventional embolization and radiation therapy may be used, but these treatments may result in several complications including destructive biliary damage and sclerosing cholangitis [15,16]. Percutaneous radiofrequency ablation therapy was considered as a minimally invasive and safe treatment for malignant neoplasms of the liver, but it has limitations for lesions near the diaphragm or in the periphery of the liver, which may result in damage to adjacent organs [17]. Microwave ablation offers all the benefits of radiofrequency ablation and some additional advantages, including reduced procedure time and decreased heat-sink effect [18]. Therefore, microwave ablation should be considered as highly effective treatment for huge hepatic hemangioma.

There have been few studies comparing the efficacy and safety of open resection and laparoscopic microwave ablation of giant hemangioma. Therefore, we conducted this retrospective study to evaluate the advantages and disadvantages of laparoscopic microwave ablation (LMWA) and conventional open resection (ORES) in the treatment of giant hemangioma. Furthermore, the technical points of laparoscopic microwave ablation were also analysed.

## 2 Methods

### 2.1 Patients

This is a single-center non-randomized retrospective study. According to the STROCSS criteria, we prospectively collected data from patients who underwent laparoscopic microwave ablation (LMWA group) or conventional open resection (ORES group) for giant hemangioma between January 1, 2014 and June 30, 2016 in authors' hospital [19]. This study was approved by the authors' institutional review board. Clinicians explained the therapeutic options to all patients. An appropriate method (surgical resection or laparoscopic microwave ablation) was selected for each patient, with informed consent for the procedure being obtained, taking into account the patient's preference and the cost, as well as the medical evidence. Written informed consent and the use of data for research purposes was obtained from all patients before each treatment (Research Registry Unique Identifying Number: researchregistry4653).

Inclusion criteria were the following: (a) definite diagnosis of a giant hemangioma (≥5 cm) based on at least two coincidental radiological findings on enhanced CT/MRI or contrast-enhanced ultrasound [1]; (b) presence of clinical symptoms that are typically caused by giant hemangioma excluding the presence of other hepatobiliary or gastrointestinal disorders; (c) patient was anxious and insisted on a surgical treatment [20].

Exclusion criteria were as follows: (a) patients who did not give informed consent; (b) patients with other types of solid liver tumors; (c) pregnant patients [21].

**Fig. 1:**
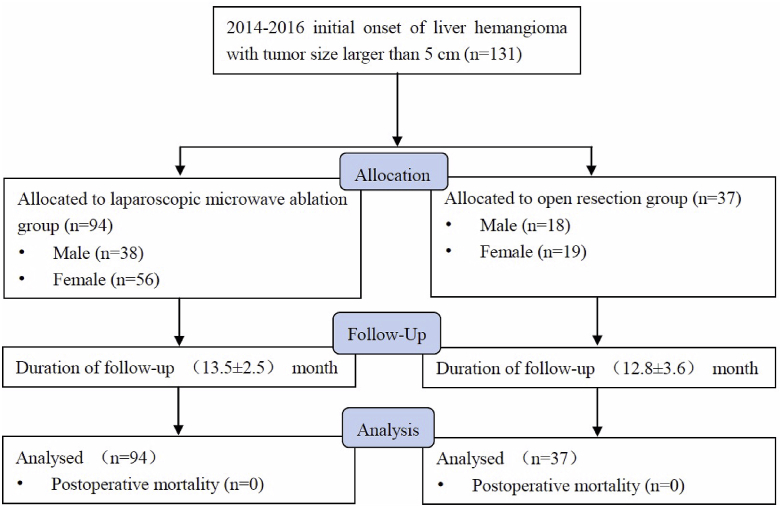
Flow diagram for the study.

131 patients with giant hemangiomas were recruited. Among them, 37 patients were treated with ORES and 94 patients chose LMWA (Fig. 1).

### 2.2 Instruments and equipment

A KY-2000 microwave multifunctional therapeutic apparatus (Kangyou Medical Instruments, Nanjing, China) was used for the ablation. This system consisted of a microwave generator with a frequency of 2450 MHz, a power output of 10–100 W and a 15-cm high-strength water-cooled antenna with an external diameter of 1.9 mm (KY2450B). Laparoscopic ultrasonography of the liver was performed using an 8820e laparoscopic ultrasound probe and the FlexFocus 800 ultrasound system (BK Medical, Herlev, Denmark).

### 2.3 Resection and ablation protocols

Surgical resection was carried out in a standard procedure by surgical team consisting of two experienced surgeons specializing in liver surgery at our hospital. Decisions concerning the extent of liver resection were based on lesion location and underlying liver status. A conventional generous right subcostal incision was made. If necessary, the pringle maneuver was used during the operation.

**Fig. 2:**
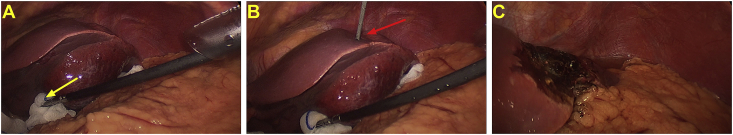
Laparoscopic view of a lesion. A. The pre-operative lesion, with wet gauze was placed between the liver and stomach to avoid surrounding tissue damage caused by the ablation thermal radiation of the ablation (yellow arrow). B. The intra-operative lesions, chosen the normal liver lesions within 0.5–1.0 cm from the edge of the tumour to start ablation (red arrow). C. The post-operative lesion, the lesion became a depressed mass with a hard texture after ablation. (For interpretation of the references to color in this figure legend, the reader is referred to the Web version of this article.)

During laparoscopic microwave ablation, patients were positioned in a supine position after the success of the general anaesthesia. In patients who had tumour locations limited to the right lobe of the liver or the posterior segments, the position was adjusted according to the lesion location. Once pneumoperitoneum was established, a 10-mm trocar was inserted in the umbilicus. An initial laparoscopic exploration of the peritoneal cavity was performed, and a second 12-mm trocar was inserted 3 cm beneath the mucrosterni. A 5-mm trocar was placed below the right or left costal margin depending on the location of the hepatic hemangiomas [8]. The laparoscopic ultrasonic probe entered the abdominal cavity through the 12-mm hole, thus further clear the location of the hemangioma and the condition of peripheral blood supply. Normal liver lesions within 0.5–1.0 cm from the edge of the tumour were chosen locations to start the ablation (Fig. 2B, red arrow). The ablation power range was 60–65 W, and the ablation time was determined according to the size of the lesion. To prevent bleeding, track ablation was performed when the antenna was withdrawn.

### 2.4 Perioperative period treatment of ablation

During the operation, any color change of the urine was monitored, conventionally, 125 mL of 5% sodium bicarbonate was used to alkalize the urine. Patients' vital signs were monitored for 12 h after surgery, and liver and kidney function and routine blood analysis were examined. The drainage tube was routinely monitored, and the abdominal cavity tube and indwelling urinary catheter remained in all patients for 24 h after the surgery. Twelve hours after the operation, the patients were allowed to have a liquid diet, and drinking as much water as possible was encouraged to promote urination.

### 2.5 Data collection

Primary hemangioma characteristics assessed were size, location and number of lesions. Data on preoperative variables were obtained including: age, gender, reasons for operation, α-fetoprotein (AFP), prothrombin time (PT), creatinine (Cr), albumin (ALB), serum markers of hepatitis, total bilirubin (TB), alanine aminotransferase (ALT) and aspartate aminotransferase (AST). Postoperative details included the type of surgical therapy, operative blood loss, intraoperative blood transfusions, operative time, length of postoperative hospital stay, month of follow-up, postoperative complications and hospital cost. ALT and AST were measured at postoperative days 1 and 3. Pain was evaluated at the 12th, 24th and 48th postoperative hour using a linear analog pain scale (visual analog scale, VAS) that ranged from 0 to 10 (0, no pain; 10, worst imaginable pain).

### 2.6 Postoperative follow-up

**Fig. 3:**
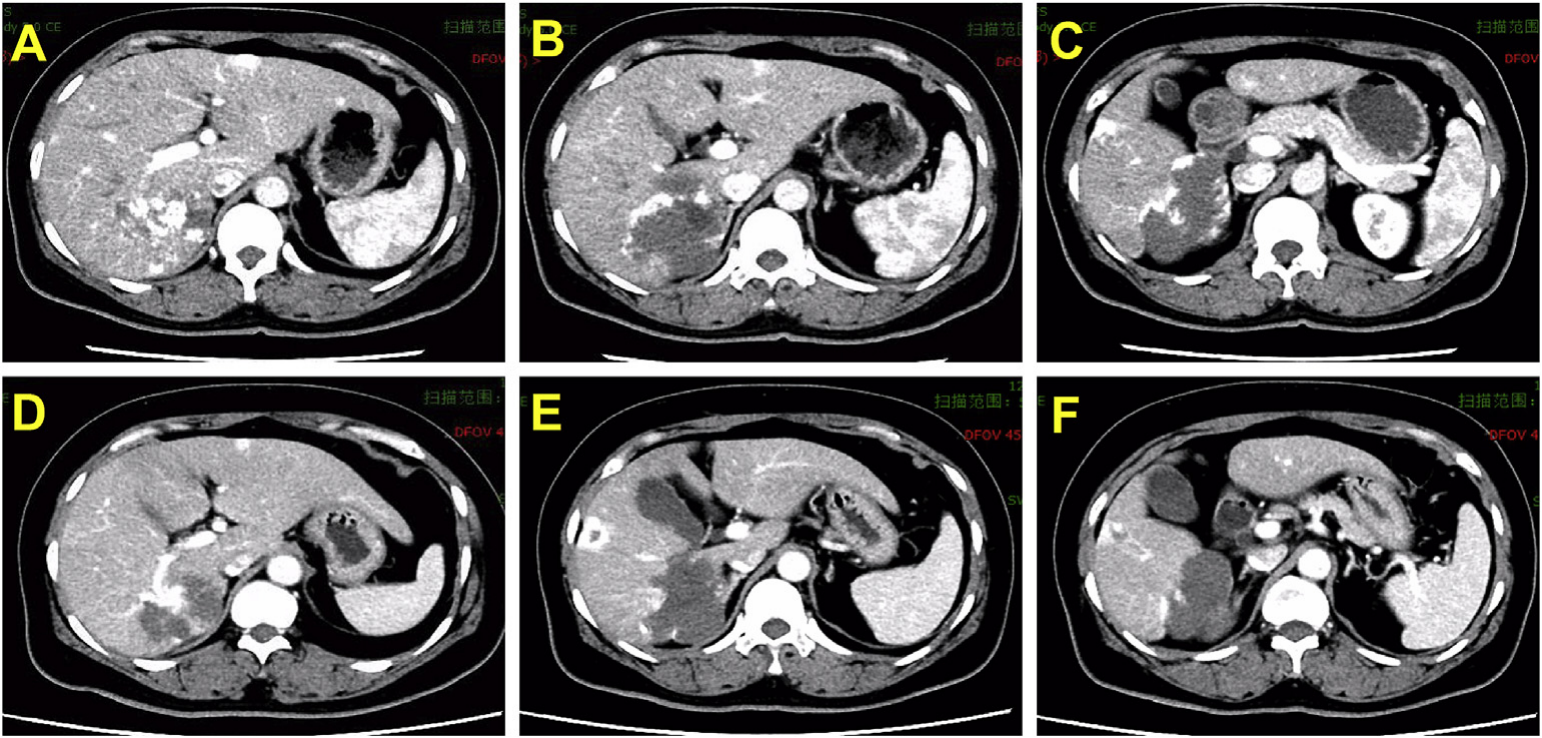
A 48-year-old woman had large hepatic hemangiomas in the right lobe (10.0 cm) as revealed by abdominal CT scans (A–C). Three months after ablation treatment, CT scans in the arterial phase showed that the hepatic hemangiomas in the right lobe had been completely ablated (D–F).

All patients underwent ultrasonography one month after operation. The post-treatment response was evaluated by enhanced CT or MRI at 3-month intervals. The lack of focal or irregular enhancement adjacent to the ablation zone on the enhanced CT or MRI scans was defined as complete ablation [22]. Follow-up was completed by either chart review or telephone interview in June 30, 2017. Typical CT images obtained during the follow-up are shown in Fig. 3.

### 2.7 Statistical analysis

Values were expressed as means ± standard deviation (SD). Continuous date were tested by using the Student *t*-test and analysis of variance. Categorical data were analysed using the χ^2^ test or Fisher exact test. *P* < 0.05 was considered statistically significant. All statistical analyses were performed with SPSS version 21.0 (SPSS Inc., Chicago, IL, USA).

## 3 Results

### 3.1 Patient characteristics

According to the patient inclusion and exclusion criteria, 131 patients were included into this study finally, with 37 patients underwent ORES and 94 patients underwent LMWA. Among them, 18 male and 19 female with an average age of 46.4 ± 12.5 years (range, 23 to 76 y) in ORES group, 38 male and 56 female with an average age of 43.2 ± 10.3 years (range, 25 to 69 y) in LMWA group. In ORES group, totally have 60 lesions, 27 patients had a solitary lesion, 2 patients had two lesions and 8 patients had multiple lesions (≥3). In LMWA group, totally have 148 lesions, 60 patients had a solitary lesion, 21 patients had two lesions and 13 patients had multiple lesions (≥3). Eleven patients were found to have additional intrahepatic lesions detected with laparoscopic ultrasound that were not detected pre-operatively in LMWA group. In ORES group, the main lesions were located in the right lobe in 14 patients and 23 in the left lobe. In LMWA group, the main lesions were located in the right lobe in 65 patients and 29 in the left lobe. Take the transverse section of the largest lesions as mean maximum diameter, the mean maximum diameter was 8.3 ± 2.7 cm (range, 5.2–15.6 cm) in ORES group and 8.5 ± 2.1 cm (range, 5.4–18.7 cm) in LMWA group. In ORES group, the reasons for operation were conventional medical examination in 19 patients, abdominal discomfort in 15 patients and enlargement of hemangioma in 3 patients. In LMWA group, the reasons for operation were conventional medical examination in 64 patients, abdominal discomfort in 21 patients and enlargement of hemangioma in 9 patients. The laboratory data were summarized in Table 1.

**Table 1:** Baseline characteristics of patients.

	LMWA (n = 94)	ORES (n = 37)	*P* value
Age (years)	43.2 ± 10.3	46.4 ± 12.5	>0.05
Gender (male/female)	38/56	18/19	>0.05
Number of lesion, n (%)			
Total number	148	60	>0.05
Single lesion	60 (63.8%)	27 (73.0%)	
Two lesions	21 (22.3%)	2 (5.4%)	
Multiple lesions	13 (13.8%)	8 (21.6%)	
Location of main lesion, n (%)			
Left lobe	29 (30.9%)	14 (37.8%)	>0.05
Right lobe	65 (69.1%)	23 (62.2%)	>0.05
Tumour size (cm), mean ± sd			
Pre-operation	8.5 ± 2.1	8.3 ± 2.7	>0.05
1 month after operation	6.3 ± 1.8	0	<0.001
6 months after operation	4.3 ± 1.5	0	<0.001
Laboratory date, mean ± sd			
TB (μmol/L)	14.5 ± 3.7	16.7 ± 2.9	<0.01
ALT (U/L)	24.3 ± 4.6	25.2 ± 4.2	>0.05
AST (U/L)	33.6 ± 5.3	35.4 ± 4.8	>0.05
ALB (g/L)	38.7 ± 4.1	40.2 ± 2.6	<0.05
PT (s)	14.1 ± 1.1	13.8 ± 1.4	>0.05
Cr (μmol/L)	65.4 ± 12.7	68.4 ± 15.6	>0.05
AFP (μg/L)	6.2 ± 2.1	6.8 ± 1.5	>0.05
Hepatitis B virus, n (%)	4 (4.3%)	2 (5.4%)	>0.05
Reasons for operation, n (%)			
Conventional medical examination	64 (68.1%)	19 (51.4%)	>0.05
Abdominal discomfort	21 (22.3%)	15 (40.5%)	<0.05
Enlargement of hemangioma	9 (9.6%)	3 (8.1%)	>0.05

### 3.2 Surgical outcomes and postoperative courses

**Fig. 4:**
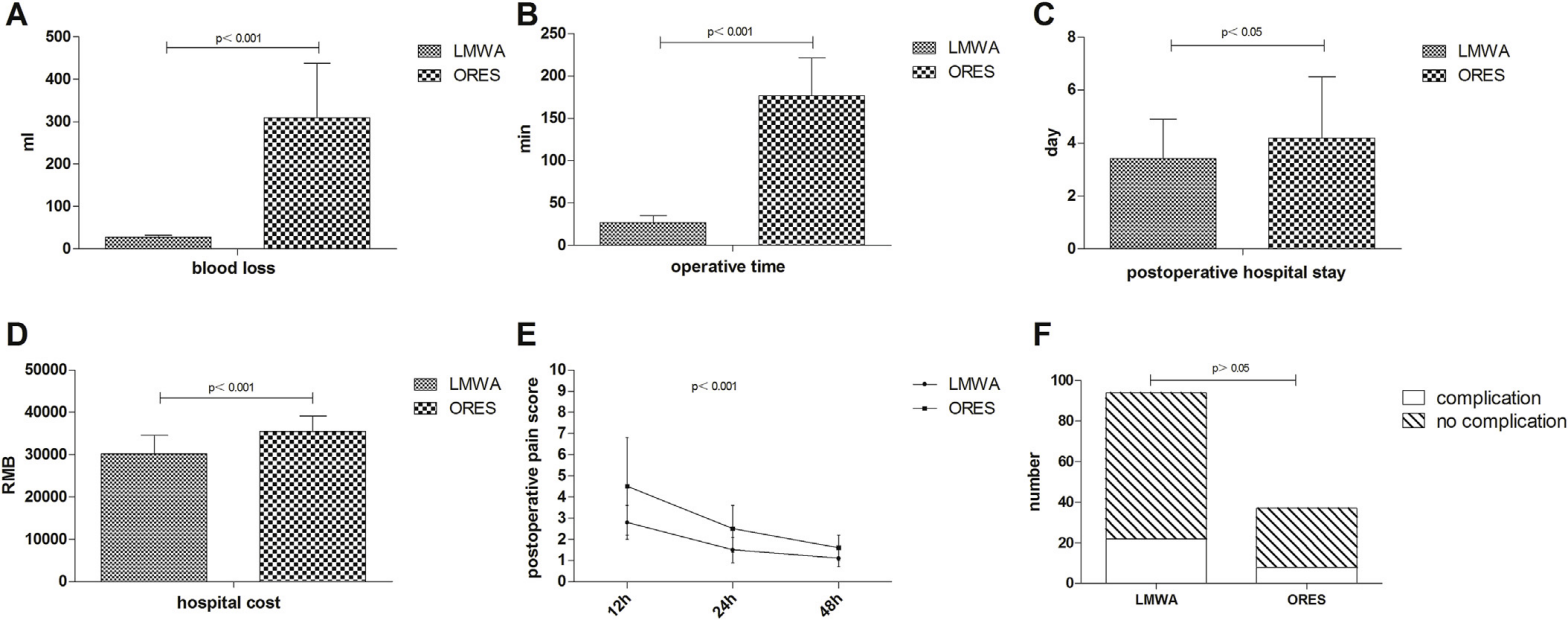
Surgical outcomes and postoperative courses.

Operations were successfully performed in all patients. The surgical outcomes and postoperative courses were summarized in Table 2. Patients in LMWA group had less blood loss (27.4 ± 4.8 mL vs. 310.4 ± 127.2 mL, *P* < 0.001, Fig. 4A) and shorter operative time (26.7 ± 8.5 min vs. 176.5 ± 44.6 min, *P* < 0.001, Fig. 4B) compared to the ORES group. Five patients required a homologous blood transfusion during surgery in ORES group. The ALT and AST levels in two groups were significantly higher than preoperative levels, but the increased ALT and AST levels in both groups declined after operation. There were no significant differences in ALT and AST levels between two groups. The postoperative hospital stay were 3.4 ± 1.5 and 4.2 ± 2.3 days, respectively (*P* < 0.05, Fig. 4C). In our current study, the hospital cost (RMB) was limited to 30143.34 ± 4358.72 in LMWA group and 35467.82 ± 3574.52 in the ORES group (*P* < 0.001, Fig. 4D). After LMWA, patients experienced significantly less pain than those patients undergoing ORES (*P* < 0.001, Fig. 4E). There were no significant differences in total complication rates between the two groups (Fig. 4F). Adverse events observed in the ORES group were: fever (8 cases), biliary leakage (1 case), wound infection (2 cases) and postoperative bleeding (1 case). Adverse events in the LMWA group included haemoglobinuria (22 cases), fever (12 cases), skin burns (4 cases) and pneumothorax (1 case).

**Table 2:** Surgical outcomes and postoperative courses.

	LMWA (n = 94)	ORES (n = 37)	*P* value
Operative blood loss (mL), mean ± sd	27.4 ± 4.8	310.4 ± 127.2	<0.001
Operative time (min), mean ± sd	26.7 ± 8.5	176.5 ± 44.6	<0.001
Blood transfusions, n (%)	0 (0%)	5 (13.5%)	<0.01
ALT (U/L), mean ± sd			
Pre-operation	24.3 ± 4.6	25.2 ± 4.2	>0.05
1 day after operation	257.4 ± 84.7	231.4 ± 24.3	>0.05
3 days after operation	108.5 ± 35.7	93.8 ± 47.6	>0.05
AST (U/L), mean ± sd			
Pre-operation	33.6 ± 5.3	35.4 ± 4.8	>0.05
1 day after operation	352.2 ± 62.4	328.8 ± 57.4	>0.05
3 days after operation	134.6 ± 56.7	118.3 ± 42.5	>0.05
Hospital cost (RMB), mean ± sd	30143.34 ± 4358.72	35467.82 ± 3574.52	<0.001
Post-operative hospital stay (day), mean ± sd	3.4 ± 1.5	4.2 ± 2.3	<0.05
Follow-up (month), mean ± sd	13.5 ± 2.5	12.8 ± 3.6	<0.05
Post-operative pain score, mean ± sd			
12 h	2.8 ± 0.8	4.5 ± 1.3	<0.001
24 h	1.5 ± 0.6	2.5 ± 1.1	<0.001
48 h	1.1 ± 0.4	1.6 ± 0.6	<0.001
Complications, n (%)			
Total number	22 (23.4%)	8 (21.6%)	>0.05
Hemoglobinuria	22 (21.6%)	0 (0%)	
Fever (body temperature >39 °C)	12 (12.8%)	8 (21.6%)	>0.05
Skin burns	4 (4.3%)	0 (0%)	
Pneumothorax	1 (1.1%)	0 (0%)	
Biliary leakage	0 (0%)	1 (2.7%)	
Wound infection	0 (0%)	2 (5.4%)	
Post-operative bleeding	0 (0%)	1 (2.7%)

### 3.3 Post-operative follow-up

At a mean follow-up period of 12.8 ± 3.6 months in ORES group and 13.5 ± 2.5 months in LMWA group, no long-term complications or hemangioma recurrence were observed. All 60 hemangiomas in the ORES group were completely removed. Of the 94 patients in LMWA group, the mean diameter of lesion zone was reduced from 8.5 ± 2.1 to 6.3 ± 1.8 cm after 1 month (*P* < 0.001) and decreased further 6 months later (to 4.3 ± 1.5 cm, *P* < 0.001, Table 1).

## 4 Discussion

Most liver hemangioma are small and asymptomatic and do not require surgical intervention. However, if the tumour reaches 5 cm or larger, it likely becomes symptomatic [2]. Several scholars have reported that liver hemangioma has a risk of rupture, and although the rupture is rare, the associated mortality rate is high [23]. Therefore, surgeons prefer to use tumour diameter greater than 5 cm, abdominal discomfort or enlargement of size as surgical indication of hemangiomas [21].

The mechanism of microwave ablation is creating a microwave electric field around a needle, causing the polar molecules in the living tissue, such as water, proteins and carbohydrates and charged particles, such as potassium, sodium and chloride ions to heat up under the action of the electromagnetic force. A large amount of heat is quickly generated and causes coagulation necrosis [24,25]. Compared with radiofrequency ablation, microwave ablation can produce larger ablation zones and hotter temperatures [26]. As hemangiomas are composed large amounts of intratumoral water, we hypothesized that microwave would be a more effective treatment.

Percutaneous ultrasonography-guided radiofrequency ablation is increasingly being used for the treatment of liver malignancies [27]. We do not choose a percutaneous approach to ablate the hemangiomas under the guidance ultrasound as the follow reasons. First, when then lesion located in subcapsular or near-gallbladder areas, there is a risk of damage to adjacent tissues [28,29]. Second, When there is a small amount of bleeding, the ultrasound is not easy to find in time. Third, it could increase the risk of abdominal adhesion. By laparoscopic cleaning and anti-adhesion treatment, this risk can be reduced [30].

In recent years, a large number of laparoscopic liver resection technologies have been developed for clinical applications [31,32]. Laparoscopy combined with microwave ablation for liver hemangioma has the advantages, including minimal trauma, rapid recovery, less bleeding and fewer complications [33]. There are few studies comparing efficacy and safety of open resection with laparoscopic microwave ablation for the management of giant hemangioma. The results of our study show that patients in LMWA group had less blood loss, lower hospital cost, shorter operative time and postoperative hospital stay, respectively. The results of our study show that laparoscopic microwave ablation is a feasible, safe and effective technique for treating giant hemangioma. To achieve good results, stricter operative indications could be recommended for laparoscopic microwave ablation. Our experiences are as follows.

The skin incision width of the puncture point was generally 1.5–2 times the diameter of the needle (approximately 3–4 mm). An ablation needle that is too narrow or too wide could lead to difficulty adjusting the needle position or unnecessary damage, respectively. If the needle position needs to be adjusted during intermittent ablation applications, the needle should be removed slowly, avoiding skin or surrounding tissue damage caused by the needle heat.

**Fig. 5:**
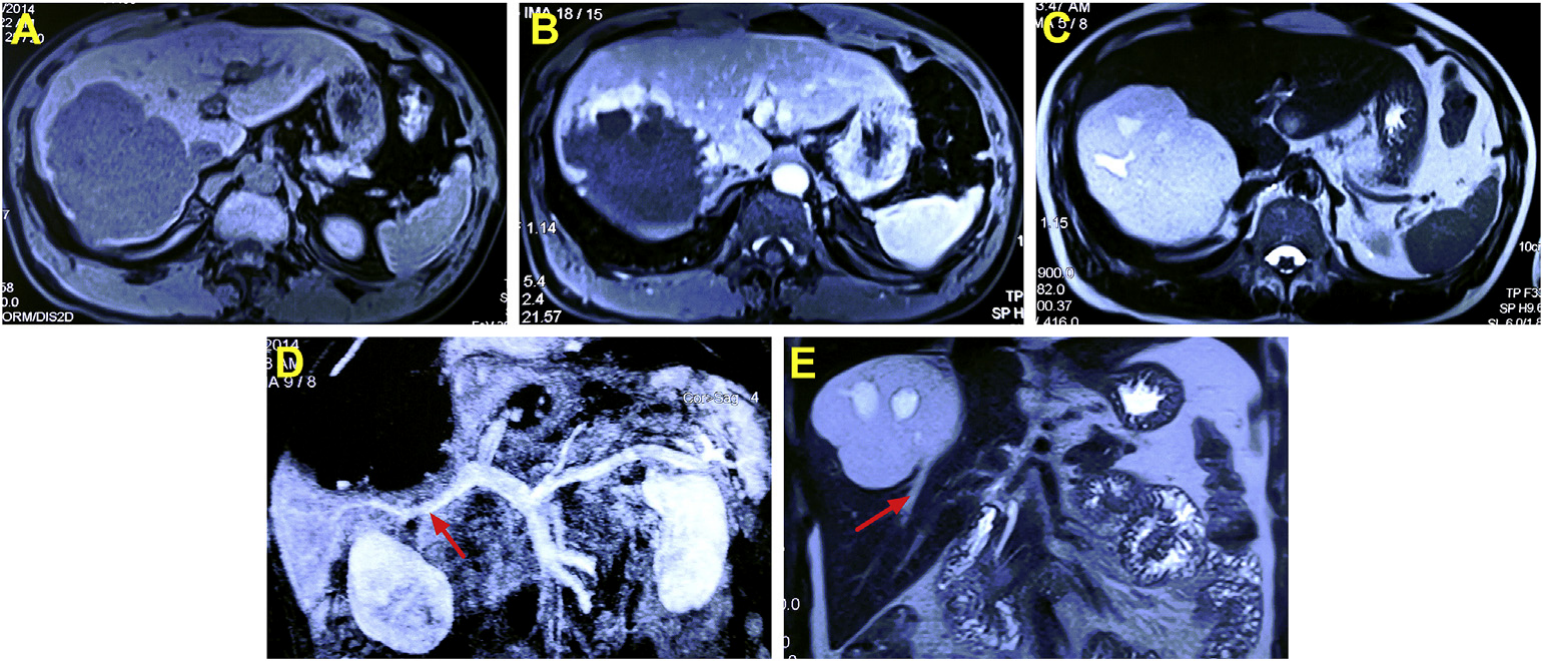
A 54-year-old man had a large hepatic hemangiomas in the right lobe (12.0 cm) as revealed by abdominal MRI scans (A–C). The ablation start from the main blood vessel in the areas of lesions (D, E, red arrow). (For interpretation of the references to color in this figure legend, the reader is referred to the Web version of this article.)

When the lesion diameter was more than 10 cm, the ablation strategy was as follows. The first porta was dissected, and the left or right hepatic artery was temporarily blocked according to the lesion location. The ablation procedure should start from main blood vessel in the areas of lesions (Fig. 5, red arrow). After the deep blood supply was blocked, the shallow area was quickly ablated [27]. The initial insertion depth should be closely monitored. The ablation strategy was to address deep lesions first, followed by slowly backing out the needle to ablate the shallow area. This technique improves the ablation efficiency and reduces the ablation time. An additional 125 mL of 5% sodium bicarbonate can be used to alkalize urine after the operation [8].

When the lesion is located in the diaphragm or adjacent to the surrounding organs, wet gauze can be placed between the gap and 50–100 mL of cold saline can be injected to avoiding surrounding tissue damage caused by thermal radiation of the ablation (Fig. 2A) [34]. Pneumothorax is a common complication when a lesion is near the diaphragm, with authors reporting an incidence rate of 15–45% [35]. Once a pneumothorax occurs, the ablation operation should be stopped immediately and the needle should be removed to avoid additional damage to the diaphragm and enlargement of the damaged area [36,37]. Because the intra-abdominal pressure is higher than the chest pressure during laparoscopic surgery, gas in the abdominal cavity can be pressed into the chest through the broken hole. Closing the pneumoperitoneum and discharging the carbon dioxide in the abdominal cavity are useful for reducing the abdominal pressure. Then, blood pressure saturation should be closely monitored through cooperation with the anesthesiologist. Sometimes, a small amount of CO_2_ can be absorbed by the body itself. Few patients need the thoracic cavity closed drainage, but the post-operative chest X-ray must be evaluated [37,38].

Some scholars advocated that for the patients whose lesions are adjacent to the gallbladder, laparoscopic cholecystectomy should be performed before ablation to avoid thermal injury to the gallbladder [8,33]. Eight patients had lesions encroaching on the gallbladder fossa, but the laparoscopic cholecystectomy was not performed. Because the liquid in the gallbladder reduces the thermal sedimentary effect by itself. The blood supply area of the hemangioma is located far from the gallbladder, so the ablation should initially start away from the gallbladder and then slowly be directed to the gallbladder. Damage to the gallbladder should be avoided as much as possible. The gallbladder itself has a small amount of activity, and we could flip the gallbladder to achieved a completely ablation. Once the part of the hemangioma far from the gallbladder is ablated and the blood vessel is solidified, the residue should not be increased and can partly be absorbed by the body itself. Low-power ablation and irrigation with cold saline to the ablation area also reduce damage to the gallbladder. Throughout the entire process, care should be taken to protect the gallbladder triangle.

Our study is limited by its retrospective nature and this is a single-center non-randomized study. The outcome of operation primarily depend on the experience of the surgeon. Since the medical classification system of China is still being ironed out, that result certain kinds of diseases abnormally concentrated in major medical institutions. The morbidity in this study is not consistent with the incidence of epidemiological morbidity. However, this would be conducive to better research on the advantages and disadvantages of different surgical methods.

In conclusion, as a minimally invasive treatment option for treating giant hepatic hemangioma, laparoscopic microwave ablation is a safe and effective therapeutic modality and its clinical application should be promoted.

## Conflicts of interest

The authors declare no conflicts of interest.

## Sources of funding

This research project was supported by the National Natural Science Foundation of China (No. 81371840).

## Ethical approval

This study was approved by the institutional review board of Union Hospital, Tongji Medical College, Huazhong University of Science and Technology, Wuhan, China.

Approve number: 2018S013.

## Research registry number

Chinese Clinical Trial Registry: ChiCTR1800018679.

Research Registry Unique Identifying Number: researchregistry4653.

## Author contribution

Lei Zhang, Libo Chen, Qinggang Hu: Study design, patient selection and evaluation, statistical analysis, manuscript review.

Min Tian, Lei Zhao: Study outcomes, statistical analysis, manuscript generation.

Lei Zhang, Jun Xiong: Patient selection and evaluation, data, recollection.

## Guarantor

Lei Zhang, Libo Chen, Jun Xiong.

## Provenance and peer review

Not commissioned, externally peer reviewed.
